# Carbon dioxide laser therapy for the management of genitourinary syndrome of menopause: A meta‑analysis of randomized controlled trials

**DOI:** 10.3892/etm.2023.12297

**Published:** 2023-11-13

**Authors:** Yihua Ni, Junyu Lian

**Affiliations:** Department of Gynecology, Fujian Maternity and Child Health Hospital, College of Clinical Medicine for Obstetrics and Gynecology and Pediatrics, Fujian Medical University, Fuzhou, Fujian 350001, P.R. China

**Keywords:** vulvovaginal atrophy, vaginal dryness, menopause, laser, genitourinary syndrome of menopause, carbon dioxide

## Abstract

Genitourinary symptoms of menopause (GSM) affect ~50% of women after menopause. Recently, CO_2_ laser therapy has been used for managing GSM but without high quality evidence. The present review assessed the effectiveness of CO_2_ laser therapy in the management of GSM. PubMed, Embase, Web of Science, CENTRAL and Scopus databases were searched for randomized controlled trials (RCTs), published up to June 30, 2023, comparing CO_2_ laser and sham laser treatments for GSM management. The outcomes of interest included Female Sexual Function Index (FSFI), Vaginal Health Index (VHI) and visual analog scale (VAS) for dyspareunia, dryness, burning, itching and dysuria. A total of seven RCTs were included in the review and meta-analysis, with 6/7 studies using three sessions of laser therapy, 4-8 weeks apart. Meta-analysis demonstrated no statistically significant difference in FSFI [mean difference (MD), -1.48; 95% CI, -5.85, 2.89; I^2^=45%] and VHI scores (MD, -0.18; 95% CI, -1.66, 1.31; I^2^ =72%) between laser and control groups. Meta-analysis also demonstrated no statistically significant difference in VAS scores for dyspareunia (MD, -1.63; 95% CI; -4.06, 0.80; I^2^=91%), dryness (MD, -1.30; 95% CI, -3.14, 0.53; I^2^=75%), burning (MD, -0.76; 95% CI, -2.03; 0.51 I^2^=56%), itching (MD, -0.28; 95% CI, -0.95, 0.38; I^2^=0%) and dysuria (MD, 0.15; 95% CI, -0.37, 0.67; I^2^=23%) between the groups. The included RCTs had low risk of bias. In conclusion, meta-analyses of high-quality sham-controlled RCTs indicated that CO_2_ may not have any beneficial effect on GSM. Limited data and high heterogeneity in meta-analyses in this area of research are important limitations that need to be addressed by future RCTs.

## Introduction

Genitourinary syndrome of menopause (GSM) is prevalent in post-menopausal women and is associated with vaginal itching, burning, dryness, dyspareunia and urinary tract dysfunction (1). GSM affects ~50% of women after menopause and leads to reduced sexual function and poor quality of life (2). GSM results from the reduction in estrogen and subsequent anatomical and functional changes in the urogenital tissues, such as reduction in vaginal blood flow, increase in pH, altered expression levels of elastin and collagen, reduction in secretions and thinning of the epithelium (1). The resulting symptoms of vaginal itching, burning, dryness, dyspareunia, painful sexual activity and urinary tract dysfunction of varying intensities are associated with a reduced quality of life (2,3).

Guidelines from the North American Menopause Society state that the initial management protocol of GSM includes vaginal moisturizers, lubricants and continuation of sexual activity (4). Lubricants are a temporary solution that are used during sexual activity to reduce tissue irritation; however, moisturizers are longer acting and aim to reduce dryness and vaginal pH thereby reducing GSM. Additionally, local estrogen therapies are also effective in managing moderate to severe cases of GSM as they specifically target the underlying pathology, namely the hypoestrogenic vaginal tissue (5). Nevertheless, local estrogen therapies have relatively low compliance (6). Topical estrogens often lead to incomplete relief of symptoms, and their effect stops with discontinuation of treatment. Therefore, other modes of therapy for this condition are needed (6).

In the past decade, laser therapy using a general fractionated CO_2_ laser has, anecdotally, become increasingly utilized in the management of GSM. CO_2_ laser therapy utilizes a gaseous medium to deliver a laser at 10,600 nm which is rapidly absorbed by water molecules to penetrate the vulvovaginal tissues (7). In a systematic review and meta-analysis of 25 studies, Filippini *et al* (8) reported that CO_2_ laser therapy was effective in alleviating GSM. However, the quality of evidence, assessed using risk of bias tools, was low to very low, as most studies were observational without any randomization. GSM is characterized by symptoms that are mainly subjective, such as itching, burning and dryness; therefore it is important that a placebo effect is negated during the assessment of the efficacy of any treatment. Therefore, the present systematic review and meta-analysis evaluated sham-controlled trials to assess the efficacy of CO_2_ laser therapy for the management of GSM.

## Materials and methods

### Search source and strategy

The present review was registered on PROSPERO (ID no. CRD42023432973). A systematic search of the literature for studies that were published from inception to June 30, 2023 was performed by two reviewers, separately. The databases examined were as follows: PubMed, Embase, Web of Science, CENTRAL and Scopus. Google Scholar was also searched for gray literature.

The inclusion of studies was based on the following Population, Intervention, Comparison, Outcome and Study type (PICOS) criteria: P, menopausal women with GSM; I, use of CO_2_ laser therapy; C, sham laser therapy; O, GSM evaluated by any standardized scale; and S, randomized controlled trials (RCTs). Non-randomized studies, studies using active treatment modality in the control group, editorials, theses, non-peer-reviewed studies and animal studies were excluded.

The search for studies was based on the following keywords: ‘menopause’; ‘genitourinary’; ‘vulvovaginal atrophy’; ‘carbon dioxide’; ‘CO_2_’; ‘laser’; and ‘randomized’. Different search strings were generated using ‘AND’ and ‘OR’. The search strings were similar across databases. Search details are listed in Table SI.

### Study selection

Two reviewers independently evaluated all the search results. First, the retrieved data was collated and deduplicated electronically using Mendeley (version 1.19.8, Elsevier). The titles and abstracts of all articles were screened to identify relevant studies based on the aforementioned inclusion criteria. The selected studies underwent full-text analysis. The reviewers screened these studies based on the eligibility criteria for further inclusion. Any disagreements were solved by discussion and consensus between the reviewers. The reference lists of the included studies were also examined to identify any other relevant articles.

### Extracted data and outcomes

The following data were extracted from the selected articles by two reviewers independently: First author; year of publication; study location; inclusion criteria; laser type; laser energy settings; number of laser sessions; sample size; participant age; years since menopause; study outcomes; and follow-up period. Study details extracted by the two reviewers were then cross-matched and any discrepancies were resolved.

### Risk of bias analysis

Risk of Bias 2 tool (The Cochrane Collaboration, release date 22 August 2019) was used for quality assessment (9). For each domain of the assessment tool, studies were marked as having a low or high risk of bias, or as having some concerns. The different domains of the tool included: randomization process; deviation from intended intervention; missing outcome data; measurement of outcomes; selection of reported results; and overall risk of bias.

### Statistical analysis

The present review was performed according to the Preferred Reporting Items for Systematic Reviews and Meta-Analyses guidelines (10). Statistical analysis was performed using Review Manager (RevMan; version 5.3; The Cochrane Collaboration). The outcomes for meta-analysis were selected based on the availability of data from ≥3 studies. Data were combined to generate a mean difference (MD) with 95% confidence intervals (CI). Results are presented in the form of forest plots. The meta-analysis was conducted using the random-effects model. Funnel plots were not generated due to a low number of studies included in the meta-analysis. Inter-study heterogeneity analysis was performed, yielding an I^2^ value, <50% suggested low heterogeneity and >50% suggested substantial heterogeneity between studies.

## Results

### Search results

**Figure 1:**
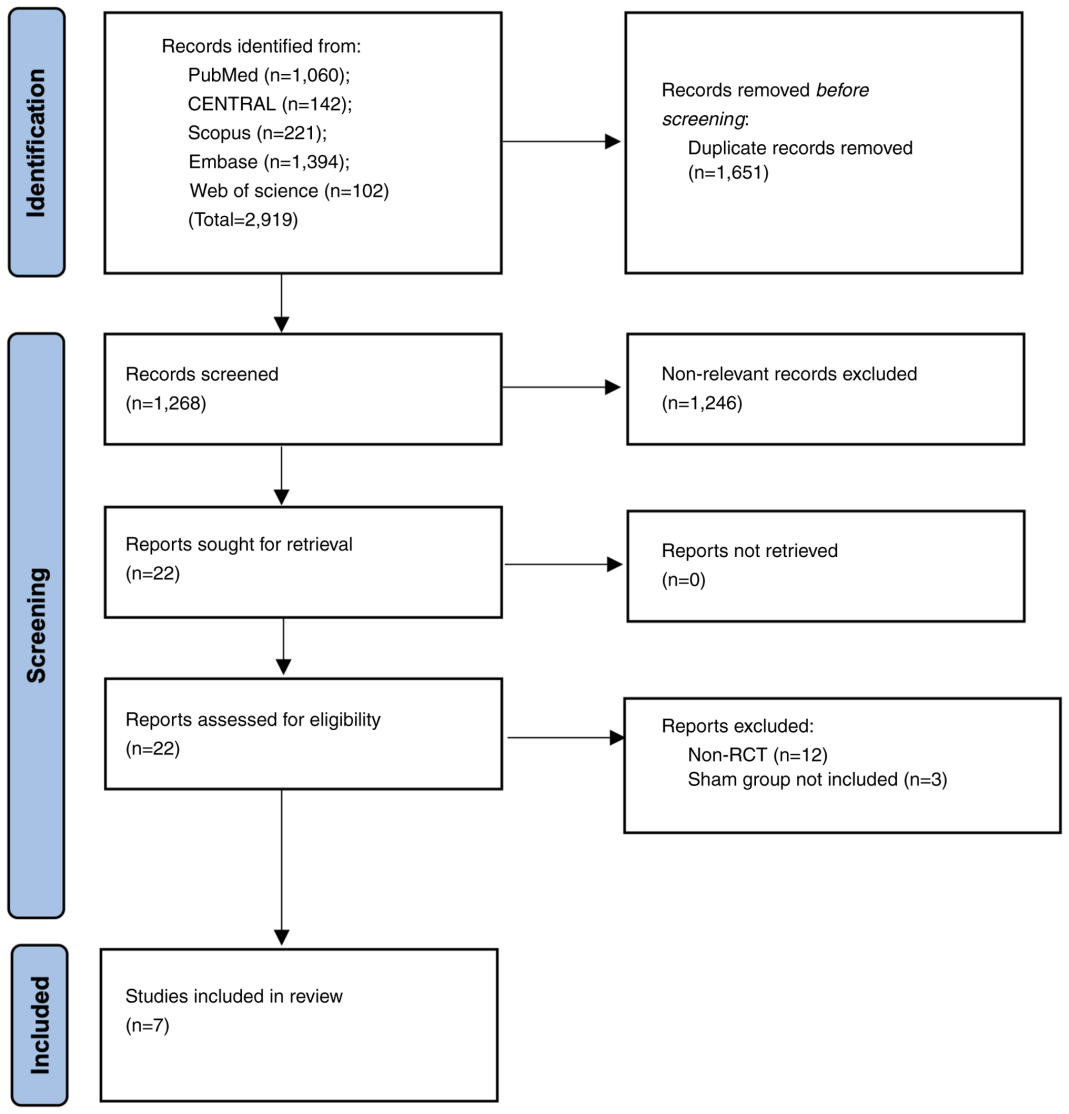
Study flowchart presenting the search strategy and number of studies at each stage. RCT, randomized controlled trial.

A total of 2,919 articles were initially retrieved. Duplicate articles were excluded and further screening was performed on 1,268 records, of which 22 studies were found to be suitable for full-text analysis. Finally, seven articles were selected for the final review and meta-analysis (11-17). The search strategy is presented in Fig. 1.

### Study details

Details of included studies are listed in Table I. All RCTs were published in the past three years and were from Thailand, Italy, Greece, United States of America, Belgium, Spain and Australia. The participants of two RCTs included only gynecological or breast cancer survivors with GSM. The remaining RCTs did not have restrictive inclusion criteria. The same fractionated CO_2_ laser equipment was used in all trials, whilst the energy output used was either 30 or 40 W. In the control groups, the same laser equipment was used as in the treatment groups, but without any laser emitted. A total of three sessions of laser therapy were used in 6/7 studies, performed 4-8 weeks apart. Only one trial used five sessions of laser therapy. There were 8-44 patients per group and follow-up was 3-6 months. The risk of bias of each study, assessed using risk of bias analysis, is presented in Table II. All included studies were determined to be high-quality RCTs with a low risk of bias.

### Meta-analysis

**Figure 2:**
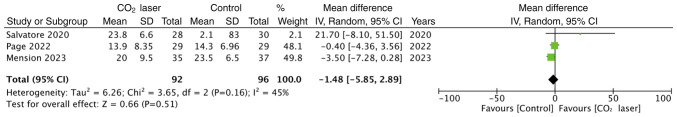
Meta-analysis of Female Sexual Function Index scores of CO_2_ laser therapy and control groups in patients with genitourinary symptoms of menopause. SD, standard deviation; IV, inverse variance.

**Figure 3:**
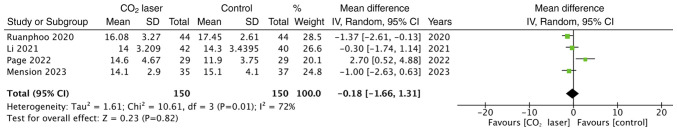
Meta-analysis of Vaginal Health Index scores of CO_2_ laser therapy and control groups in patients with genitourinary symptoms of menopause. SD, standard deviation; IV, inverse variance.

**Figure 4:**
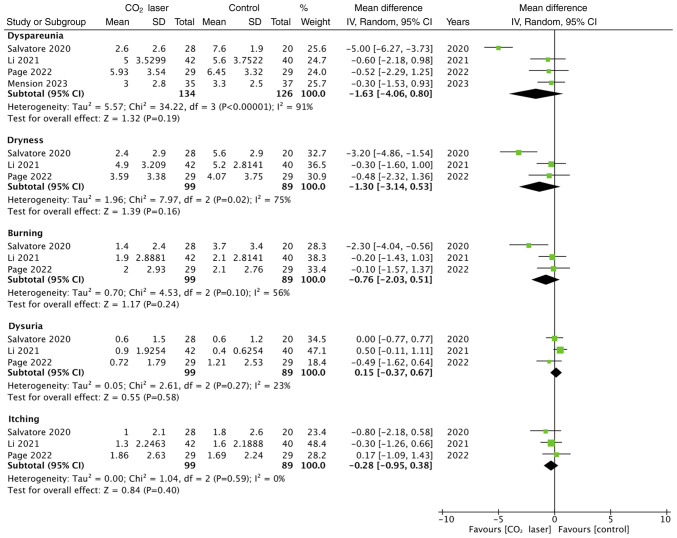
Meta-analysis of visual analog scale scores for dyspareunia, dryness, burning, itching and dysuria for CO_2_ laser therapy and control groups in patients with genitourinary symptoms of menopause. SD, standard deviation; IV, inverse variance.

The study outcomes selected for quantitative analysis, based on the availability of data, were Female Sexual Function Index (FSFI), Vaginal Health Index (VHI) and visual analog scale (VAS) for dyspareunia, dryness, burning, itching and dysuria. A total of three studies reported a final FSFI score at follow-up. Meta-analysis demonstrated there was no statistically significant difference in FSFI scores between laser and control groups (Fig. 2). Additionally, two studies only reported changes in FSFI scores and therefore, their results were not included in the meta-analysis. Quick *et al* (11) reported significantly improved FSFI scores in the laser group compared with the control group (P=0.02), whilst Cruff and Khandwala (15) reported no significant difference in FSFI score changes between the two groups (P=0.77). A total of four studies reported data on VHI, however the pooled analysis demonstrated no statistically significant difference in VHI scores between laser and sham groups (Fig. 3). The meta-analysis also demonstrated no statistically significant difference in VAS scores for dyspareunia (n=4), dryness (n=3), burning (n=3), itching (n=3) and dysuria (n=3) between the laser and control groups (Fig. 4).

## Discussion

In 2013, the North American Menopause Society (18) recommended the administration of systemic estrogen or local low-dose estrogen for the management of moderate-to-severe or mild-unresponsive GSM. Nevertheless, certain women with GSM decline the use of these therapies due to fear of side effects (such as stress incontinence), compliance issues, inadequate efficacy and contraindications (19). In the past decade, traditional therapies for GSM such as topical estrogens have begun to be substituted with innovations such as CO_2_ laser therapy which was first introduced in 2014(7). Previous studies reported that CO_2_ laser treatment was associated with certain histological changes in the vulvovaginal tissues which could potentially alter the severity of GSM (20). Zerbinati *et al* (21) reported that CO_2_ laser therapy restored the thick vaginal epithelial lining, increased collagen and ground substance in the lamina propria and increased the vascular supply of the tissue. The fractional CO_2_ laser mode of action is based on the production of heat by vaporization of water present in the cells of deeper lamina propria. The energy of the laser is precisely directed to avoid damage to the surrounding tissues. As a result of this hyper-regulated injury, there is neoangiogenesis and neocollagenesis which could improve the vaginal environment and GSM symptoms (22).

CO_2_ laser therapy has been used for GSM (8), however there is still a lack of high-quality evidence to guide clinical practice. In 2018, the United States Food and Drug Administration stated that there was inadequate data to recommend laser therapies for the optimization of sexual function and reduction of symptoms of GSM (23). Most of the data for the use of CO_2_ laser therapy is from observational studies which have a high risk of bias (8). A large meta-analysis of 25 such studies (8) reported that CO_2_ laser therapy significantly reduced symptoms of dryness (MD, -5.15; 95% CI, -5.72, -4.58), dyspareunia (MD, -5.27; 95% CI, -5.93, -4.62), itching (MD, -2.75; 95% CI, -4.0, -1.51), burning (MD, -2.66; 95% CI, -3.75, -1.57) and dysuria (MD, -2.14; 95% CI, -3.41, -0.87) in patients with GSM. Moreover, the FSFI score was significantly improved. However, the non-randomization of study participants and lack of blinding of participants and outcome assessors generated bias which hampered the interpretation and acceptance of such results (24). Another recent narrative review by D'Oria *et al* (25) reported that CO_2_ laser therapy was an effective and safe therapeutic option for treatment of vulvovaginal atrophy in gynecological cancer survivors. However, only nine studies were evaluated and quantitative synthesis was not performed. Furthermore, Khamis *et al* (26) pooled data from three sham-controlled trials and reported that CO_2_ laser therapy resulted in significant improvements in VAS score, FSFI and patient satisfaction in patients with GSM. The low number of trials included failed to generate adequate outcome data and provide high quality evidence, despite only evaluating RCTs.

Therefore, the present review provided higher quality evidence for the efficacy of CO_2_ laser therapy in the management of GSM. As, to the best of our knowledge, this is the first review that has assessed the efficacy of CO_2_ laser for GSM by using pooled analysis of only high-quality sham-controlled RCTs. The present study did not include trials that used an active comparator or placebo, namely no laser in the control group. All patients in control groups were blinded and the same laser equipment with no energy settings was applied for the same duration for all participants. Thus, the placebo effect was well-controlled in these trials (24). Additionally, all trials were blinded for outcome assessment to reduce bias in the results. Scores reported by ≥3 studies in a meta-analysis were combined to evaluate the efficacy of CO_2_ laser therapy, from which it was demonstrated that this treatment modality did not result in any significant differences in outcomes in patients with GSM. There was no statistically significant difference in total FSFI, VHI and VAS scores for dyspareunia, dryness, burning, itching and dysuria.

However, the participants of two of the included trials included only cancer survivors with GSM. Endocrine therapies used for the treatment of breast and gynecological cancer often result in adverse events including sexual dysfunction. Patients report problems with sexual desire, interest, arousal, orgasm and genitopelvic pain, and these symptoms are often underdiagnosed and undertreated (27). Patients are often treated using vaginal lubricants, moisturizers, estrogen, dehydroepiandrosterone, ospemifene and counseling, but with limited effects. It has been previously reported that combination therapies may be more beneficial in this subgroup of patients (28). Moreover, given the scarce evidence for CO_2_ laser therapy for the management of GSM in cancer survivors, there is a need for further trials focused specifically on this cohort (27,28).

The trials included in the present review did not report any major adverse events associated with the use of CO_2_ lasers. A previous study also reported that CO_2_ lasers are safe and are associated with minimal complications (29). The Manufacturer and User Facility Device Experience database, which monitors laser-based adverse events for vaginal rejuvenation, reported that pain, numbing and burning are the most common adverse effects of CO_2_ laser therapy (30). Nevertheless, in certain patients, CO_2_ laser therapy can cause serious complications, such as fibrosis, scarring, agglutination and penetrating injury. These outcomes need to be assessed in future trials (29).

There are certain limitations to the present review and meta-analysis. The number of RCTs included (n=7) was low with variations in the outcome scores and only three or four studies included in each meta-analysis. Additionally, the heterogeneity in four of the meta-analyses was high. This could be due to variations in the severity of baseline patient symptoms, differences in patient inclusion criteria and the protocol of CO_2_ laser sessions. However, due to the small number of studies in the meta-analysis, the source of the heterogeneity could not be evaluated using subgroup or meta-regression analysis. Moreover, all trials reported only short-term follow-up data (<1 year). The potential long-term benefits of CO_2_ laser therapy for the management of GSM are still unknown.

In conclusion, the present meta-analysis of high-quality sham-controlled randomized trials demonstrated that CO_2_ laser treatment may not have any beneficial effect on GSM. The present meta-analysis and qualitative analysis failed to demonstrate any significant effect of CO_2_ laser therapy on GSM, with no significant difference in FSFI, VHI and VAS scores for dyspareunia, dryness, burning, itching and dysuria with the use of a CO_2_ laser. The limited data and high heterogeneity in meta-analyses in this area of research are important limitations that need to be addressed by future RCTs.

## Supplementary Material

Key words and search strings used in the search for studies.

## Figures and Tables

**Table I:** Characteristics of the randomized control trials included in the review and meta-analysis.

First author/s, year	Country	Inclusion criteria	CO_2_ laser	No. of laser sessions	Control	Groups	Sample size	Age, years mean ± standard deviation or median (range)	Post-menopause, years	Follow-up, months	(Refs.)
Mension *et al*, 2023	Spain	Breast cancer survivors >30 years receiving aromatase inhibitors; menopause, signs or symptoms of GSM with dyspareunia, and vaginal pH ≥5; and sexually active	SmartXide2 V2LR Monalisa Touch; 40 W power, 1,000 µs dwell time	5, 1 month apart	Sham laser	Laser	35	52.3±8.3^a^	NR	6	(14)
						Control	37				
Page *et al*, 2022	Belgium	Moderate to severe symptoms of GSM (namely. vaginal dryness, vaginal itching, vaginal burning, dyspareunia and dysuria) shown by an MBS score of ≥2	SmartXide2 V2LR Monalisa Touch; 30 W power, 1,000 µs dwell time	3, 4 weeks apart	Sham laser	Laser	29	57.4±7.1	7.3±5.2	3	(12)
						Control	29	56.2±6.3	6.4±5.6		
Quick *et al*, 2021	USA	Gynecologic cancer survivors with GSM	SmartXide2 V2LR Monalisa Touch; 30 W power, 1,000 µs dwell time	3, 1 month apart	Sham laser	Laser	10	56.0±11.2	NR	4	(11)
						Control	8	56.8±6.0			
Li *et al*, 2021	Australia	Symptomatic amenorrheic for ≥12 months	SmartXide2 V2LR Monalisa Touch; 40 W power, 1,000 µs dwell time	3, 4-8 weeks apart	Sham laser	Laser	43	55±7	8 (4-14)	6	(13)
						Control	42	58±8	6 (3-9)		
Cruff and Khandwala, 2021	USA	Menopausal women (or status-post bilateral oophorectomy) with dyspareunia or vaginal dryness rated as moderate-severe, who were desirous of sexual function	SmartXide2 V2LR Monalisa Touch; 30W power, 1,000 µs dwell time	3, 6 weeks apart	Sham laser	Laser	16	61 (54-66)	14 (5-24)	6	(15)
						Control	14	59 (56-65)	10 (4-15)		
Salvatore *et al*, 2020	Greece, Italy	Postmenopausal women with GSM diagnosis according to the International Society for the Study of Women's Sexual Health and The North American Menopause Society definitions	SmartXide2 V2LR Monalisa Touch; 30 W power, 1,000 µs dwell time	3, 1 month apart	Sham laser	Laser	28	57.0±6.9	8.2±4.9	4	(16)
						Control	30	58.4±6.0	8.7±4.9		
Ruanphoo and Bunyavejchevin, 2020	Thailand	Postmenopausal women with moderate to severe intensity of any vaginal atrophy symptom	SmartXide2 V2LR Monalisa Touch; 40 W power, 1,000 µs dwell time	3, 4 weeks apart	Sham laser	Laser	44	48.9±3.0	NR	3	(17)
						Control	44	49.5±3.9			

^a^Entire sample. MBS, most bothersome symptom; GSM, genitourinary symptoms of menopause; NR, not reported.

**Table II:** Risk of bias analysis of the randomized control trials included in the review and meta-analysis.

	Risk						
First author/s, year	Randomization process	Deviation from intended intervention	Missing outcome data	Measurement of outcomes	Selection of reported results	Overall risk of bias	(Refs.)
Mension *et al*, 2023	Low	Low	Low	Low	Low	Low	(14)
Page *et al*, 2022	Low	Low	Low	Low	Low	Low	(12)
Quick *et al*, 2021	Low	Low	Low	Low	Low	Low	(11)
Li *et al*, 2021	Low	Low	Low	Low	Low	Low	(13)
Cruff and Khandwala, 2021	Low	Low	Low	Low	Low	Low	(15)
Salvatore *et al*, 2020	Low	Low	Low	Low	Low	Low	(16)
Ruanphoo and Bunyavejchevin, 2020	Low	Low	Low	Low	Low	Low	(17)

## Data Availability

The datasets used and/or analyzed during the current study are available from the corresponding author on reasonable request.
