# Supplementary material for: Carbon dioxide laser therapy for the management of genitourinary syndrome of menopause: A meta‑analysis of randomized controlled trials
Source: Exp Ther Med. 2023 Nov 13;27(1):10. doi: 10.3892/etm.2023.12297 (PMC10785041; doi:10.3892/etm.2023.12297)
Supplement: Key words and search strings used in the search for studies. [file Supplementary_Data.pdf]

Table SI. Key words and search strings used in the search for studies.

Query	Search Details
((menopause) AND (genitourinary)) AND (CO2)	("menopause"[MeSH Terms] OR "menopause"[All Fields] OR "menopausal"[All Fields] OR "menopaused"[All Fields] OR "menopauses"[All Fields]) AND ("urogenital system"[MeSH Terms] OR ("urogenital"[All Fields] AND "system"[All Fields]) OR "urogenital system"[All Fields] OR "genitourinary"[All Fields]) AND "CO2"[All Fields]
((menopause) AND (genitourinary)) AND (carbon dioxide)	("menopause"[MeSH Terms] OR "menopause"[All Fields] OR "menopausal"[All Fields] OR "menopaused"[All Fields] OR "menopauses"[All Fields]) AND ("urogenital system"[MeSH Terms] OR ("urogenital"[All Fields] AND "system"[All Fields]) OR "urogenital system"[All Fields] OR "genitourinary"[All Fields]) AND ("carbon dioxide"[MeSH Terms] OR ("carbon"[All Fields] AND "dioxide"[All Fields]) OR "carbon dioxide"[All Fields])
((menopause) AND (genitourinary)) AND (laser)	("menopause"[MeSH Terms] OR "menopause"[All Fields] OR "menopausal"[All Fields] OR "menopaused"[All Fields] OR "menopauses"[All Fields]) AND ("urogenital system"[MeSH Terms] OR ("urogenital"[All Fields] AND "system"[All Fields]) OR "urogenital system"[All Fields] OR "genitourinary"[All Fields]) AND ("laser s"[All Fields] OR "lasers"[MeSH Terms] OR "lasers"[All Fields] OR "laser"[All Fields] OR "lasered"[All Fields] OR "lasering"[All Fields])
((((genitourinary) OR (vulvovaginal atrophy)) OR (menopause)) AND (laser)) AND (randomised)	("urogenital system"[MeSH Terms] OR ("urogenital"[All Fields] AND "system"[All Fields]) OR "urogenital system"[All Fields] OR "genitourinary"[All Fields] OR (("vulvovaginal"[All Fields] OR "vulvovaginitis"[MeSH Terms] OR "vulvovaginitis"[All Fields]) AND ("atrophie"[All Fields] OR "atrophy"[MeSH Terms] OR "atrophy"[All Fields] OR "atrophied"[All Fields] OR "atrophies"[All Fields] OR "atrophying"[All Fields])) OR ("menopause"[MeSH Terms] OR "menopause"[All Fields] OR "menopausal"[All Fields] OR "menopaused"[All Fields] OR "menopauses"[All Fields])) AND ("laser s"[All Fields] OR "lasers"[MeSH Terms] OR "lasers"[All Fields] OR "laser"[All Fields] OR "lasered"[All Fields] OR "lasering"[All Fields]) AND ("random allocation"[MeSH Terms] OR ("random"[All Fields] AND "allocation"[All Fields]) OR "random allocation"[All Fields] OR "randomization"[All Fields] OR "randomized"[All Fields] OR "random"[All Fields] OR "randomisation"[All Fields] OR "randomisations"[All Fields] OR "randomise"[All Fields] OR "randomised"[All Fields] OR "randomising"[All Fields] OR "randomizations"[All Fields] OR "randomize"[All Fields] OR "randomizes"[All Fields] OR "randomizing"[All Fields] OR "randomness"[All Fields] OR "randoms"[All Fields])
